# Association Between Tissue Accumulation of Skin Autofluorescence, Disease, and Exercise Capacity in Older Adults

**DOI:** 10.3390/ijms26072913

**Published:** 2025-03-23

**Authors:** Jun-Young Sung, Jiyoun Kim

**Affiliations:** Department of Exercise Rehabilitation, Gachon University, 191 Hambakmoe-ro, Yeonsu-gu, Incheon 21936, Republic of Korea; sjy7067@gmail.com

**Keywords:** aging, skin autofluorescence, diabetes, exercise capacity

## Abstract

As a noninvasive marker of the accumulation of advanced glycation end products, skin autofluorescence is a cost-effective alternative to traditional measurement methods. This study aimed to explore the associations of skin autofluorescence levels with disease, body composition, and physical function in older Korean adults. The study included 565 older participants (129 men and 436 women) registered across 12 institutions over 3 months in Incheon, South Korea. The noninvasive evaluation of skin autofluorescence was performed utilizing an advanced glycation end product reader mu. Skin autofluorescence, glycated hemoglobin, body composition, and fitness factors were measured. Statistical significance was set at *p* < 0.05. We confirmed that skin autofluorescence levels were affected by age (>3.4, *p* < 0.001), metabolic diseases such as diabetes and hypertension (>3.4, *p* = 0.038), and fitness factors (>3.4, *p* = 0.035). The fitness factors, which also represented a major indicator of sarcopenia, were found to have a particularly pronounced effect. Our results showed the relationships between skin autofluorescence levels, disease, and fitness factors. We also found that skin autofluorescence may play a role in the expression and measurement of sarcopenia. However, further studies are warranted to validate these results in other populations and establish a clear baseline value for skin autofluorescence levels in South Korea.

## 1. Introduction

Skin autofluorescence (SAF) is a method for measuring advanced glycation end products (AGEs) using the fluorescence properties of the skin [1]. SAF has advantages such as being noninvasive, allowing for the analysis of AGE accumulation in tissues, and being low cost compared to hematologic measurements or skin biopsies [2].

AGEs denote a category of compound generated through non-enzymatic processes, wherein they form bonds between reducing sugars and free amino groups present in proteins, lipids, or nucleic acids [3]. The accumulation of AGEs in the body occurs owing to factors including dietary intake, aging, and tobacco consumption. Conditions of hyperglycemia, such as those observed in diabetes mellitus (DM), and instances of inflammation and oxidative stress accelerate the process of AGE accumulation [4]. AGEs are biomolecules that form through the glycosylation of proteins, lipids, and nucleic acids via the Maillard reaction under conditions such as DM, hyperlipidemia, and oxidative stress [5]. The initial phase of the Maillard reaction involves the interaction between the carbonyl groups of sugars and the amino-terminal groups of proteins, lipids, or nucleic acids. It can also be generated during the degradation of proteins or lipids. Additionally, AGEs can be introduced exogenously through dietary intake, particularly via the consumption of animal-based foods or foods subjected to heating or smoking processes, which are characteristic of the Western diet [6]. In addition, AGEs have been shown to cause structural modifications of proteins, potentially leading to physiological dysfunction in multiple organ systems [7].

Thus, it is crucial to determine the relationship between AGEs and SAF and determine the relationship between AGEs and degenerative diseases. Prior research on AGEs and physical function has indicated that individuals with elevated levels of the AGE carboxymethyl lysine tend to exhibit diminished grip strength and slow walking speeds [8,9,10]. Also, SAF is a tool that enables AGE measurement without hassle in the field. However, there is a lack of AGE-related research worldwide, and there are currently no studies available in the literature that have analyzed metabolic diseases, physical activities, and AGEs and SAF in older Asian populations, specifically the Korean population.

Therefore, in this study, the study was designed to confirm the applicability of SAF in the clinical field and to evaluate factors related to elderly health using AGEs. The purpose of this study was to analyze metabolic diseases, body composition, and physical function according to SAF levels in Koreans over 60 years of age, and to identify physiological relationships that may be useful in the fields of medical and elderly welfare.

## 2. Results

The physical characteristics of the participants are presented in Table 1.

**Figure 1:**
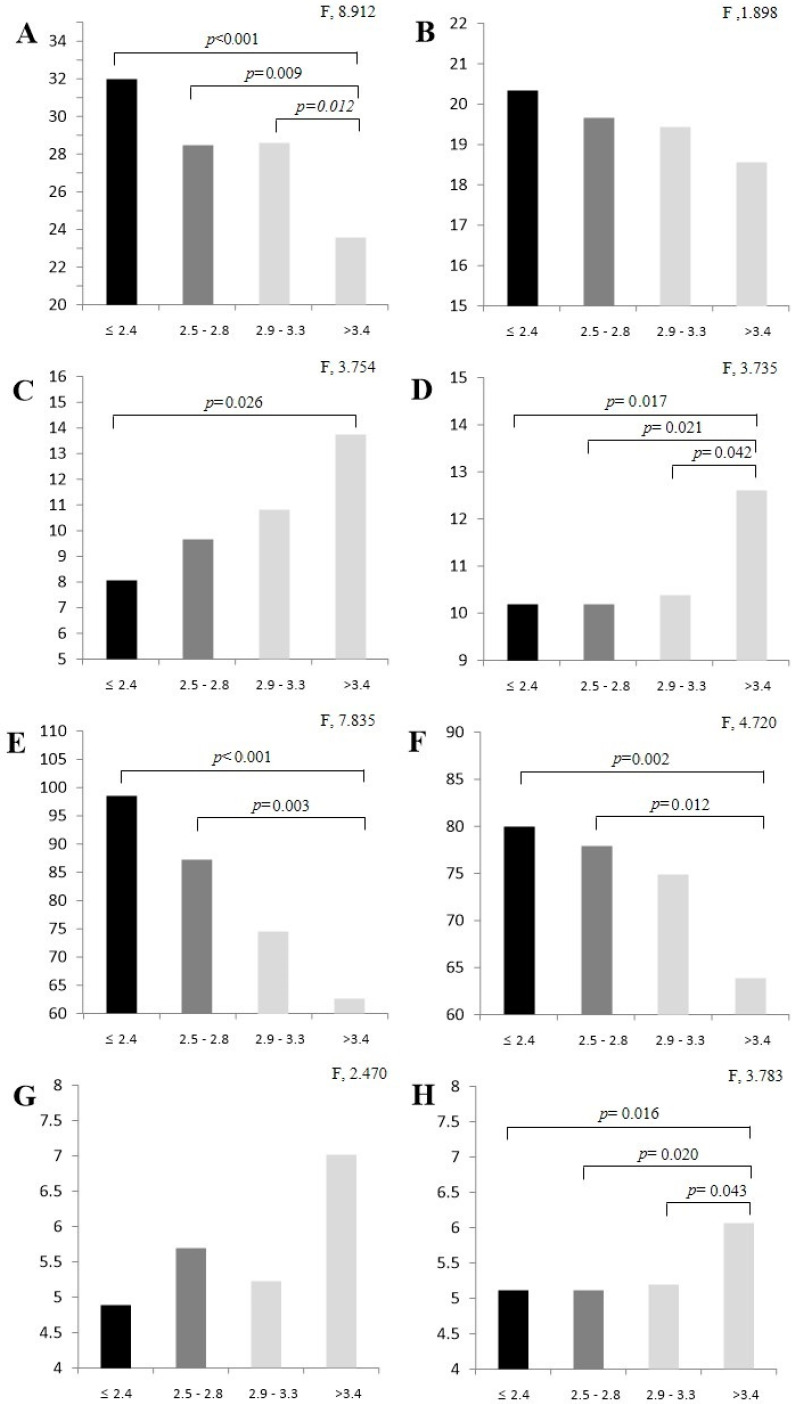
Fitness factor analysis results by SAF level: (**A**) dominant hand grip (male); (**B**) dominant hand grip (female); (**C**) timed up-and-go (male); (**D**) timed up-and-go (female); (**E**) 2 min walking test (male); (**F**) 2 min walking test (female); (**G**) gait speed (male); (**H**) gait speed (female). Values are expressed as M ± SD by Tukey test. SAF, skin autofluorescence.

The one-way analysis of variance (ANOVA) results for SAF level and fitness factor are shown in Figure 1. Men had significantly different results in terms of dominant hand grip (DHG) strength (≤2.4 vs. >3.4, *p* < 0.001; 2.5 vs. >3.4, *p* = 0.009; 2.9 vs. >3.4, *p* = 0.012), timed up-and-go (TUG; ≤2.4 vs. >3.4, *p* = 0.026), and 2 min walking test (≤2.4 vs. >3.4, *p* < 0.001; 2.5 vs. >3.4, *p* = 0.003). One-way ANOVA revealed that women had significantly different results in terms of TUG (≤2.4 vs. >3.4, *p* = 0.017; 2.5 vs. >3.4, *p* = 0.021; 2.9 vs. >3.4, *p* = 0.042), 2 min walking test (≤2.4 vs. >3.4, *p* = 0.002; 2.5 vs. >3.4, *p* = 0.012), and gait speed (≤2.4 vs. >3.4, *p* = 0.016; 2.5 vs. >3.4, *p* = 0.020; 2.9 vs. >3.4, *p* = 0.043).

The results of our principal component analysis through multiple logistic regression are shown in Table 2, which revealed that men had significantly different results in terms of calf circumference (CC; 2.5–2.8, *p* = 0.043; 2.9–3.3, *p* = 0.017; >3.4, *p* = 0.034), DHG strength (>3.4, *p* = 0.009), short physical performance battery (SPPB; 2.5–2.8, *p* = 0.013; >3.4, *p* = 0.015), TUG (2.5–2.8, *p* = 0.017; 2.9–3.3, *p* = 0.026), and 2 min walking test (>3.4, *p* = 0.022). Principal component analysis revealed that women showed significant differences in terms of glycated hemoglobin (HbA1c; >3.4, *p* < 0.001), DHG (2.5–2.8, *p* = 0.032), SPPB (2.5–2.8, *p* < 0.001; 2.9–3.3, *p* < 0.001; >3.4, *p* < 0.001), and 2 min walking test (2.9–3.3, *p* = 0.035; >3.4, *p* = 0.002). Regarding fitness-related factors such as CC, SPPB, DHG, and 2 min walking test, men tended to score higher than women.

Table 3 shows the results of the analysis performed by the model we built based on multiple logistic regression. This analysis revealed significant results for Model 1, which was adjusted for age and sex (>3.4, *p* < 0.001); Model 2, which was further adjusted for disease-related factors (>3.4, *p* = 0.038); and Model 3, which was further adjusted for fitness-related factors (>3.4, *p* = 0.035). In particular, it was confirmed that the key responding variables increased step-by-step in all figures created using Model 2; however, no significant differences were found.

## 3. Discussion

SAF is recognized as a reliable indicator of AGE accumulation in vivo. Prior investigations have demonstrated its utility as a prognostic marker for cardiovascular events and mortality risk factors in individuals with cardiovascular disease, diabetes, chronic kidney disease, and peripheral arterial disease [11]. Moreover, research has underscored a correlation between SAF and the mean levels of HbA1c, with this correlation strengthening particularly when HbA1c measurements span an extended duration [12].

In this study, as a result of measuring HbA1c levels via SAF, it was confirmed that HbA1c levels increased along with SAF measurements. Through multiple logistic regression analysis, we confirmed that the risk of disease increased as the SAF category increased in the model adjusted for disease-related factors in each SAF category. Januszewski et al. [13] and Yozgatli et al. [14] reported that SAF can serve as an independent risk factor for mortality in patients with type 1 or 2 diabetes, which is consistent with the findings of this study.

The elevation of serum AGEs and SAF, along with the onset of type 2 diabetes, can lead to mitochondrial dysfunction and heightened oxidative stress [15]. This increase in oxidative stress is associated with a greater risk of sarcopenia, which is inversely related to skeletal muscle mass and correlates with elevated levels of myokines, including proinflammatory interleukin-6 (IL-6) and tumor necrosis factor-α (TNF-α) [16]. Notably, IL-6 produced by muscle tissue differs from the proinflammatory cytokines generated by macrophages and plays multiple roles in the regulation of glucose and lipid metabolism. During physical activity, particularly under conditions of low muscle glycogen, muscle contraction stimulates an increase in IL-6 production [17,18]. This process activates AMP-activated protein kinase (AMPK) and phosphoinositide-3 (PI-3) kinase, which facilitates enhanced glucose uptake through the translocation of glucose transporter-4 (GLUT-4) to the cell membrane, thereby promoting glucose absorption and stimulating hepatic glucose production [19].

Hyperglycemia triggers an excessive generation of superoxide anions through various mechanisms. The uptake of glucose enhances the binding of nuclear factor kappa B (NFκB) in mononuclear cells, resulting in significant muscle damage in experimental models [20]. This damage is associated with the increased expression of proteasome subunits C2 and C9, as well as the Muscle RING-finger protein-1 (MuRF1) gene [21]. Additionally, insulin plays a role in reducing proteasome catalytic activity within muscle tissue. In states of insulin resistance, elevated plasma glucose concentrations can contribute to muscle atrophy.

SAF can also be used as a predictor of chronic and degenerative diseases other than diabetes. In this study, for example, significant results were obtained in models that correlated SAF with hyperlipidemia and hypertension, in addition to diabetes. This confirmation may, therefore, provide an opportunity for clinicians to expand the use of SAF for predicting other diseases as well. For example, we hypothesized that SAF may be useful in predicting sarcopenia, a degenerative muscular disease.

As the general population ages, sarcopenia emerges as a significant health concern [22]. CC, one of the main metrics of sarcopenia, has been widely used in several studies [23]. In this study, differences in CC were only confirmed in men when we examined CC changes through SAF measurements. The women exhibited no significant differences in terms of additive skeletal muscle (ASM) analysis. Regarding other key indicators of sarcopenia, such as TUG, gait speed, SPPB, and other fitness factors, the women showed results similar to those of the men.

Chen et al. [24] demonstrated an independent association between sarcopenia and body mass index (BMI), as well as CC. They reported that obesity is the most important characteristic that can affect the relationship between BMI and sarcopenia and can reduce the accuracy of predicted sarcopenia in an individual. The overweight women in this study also showed decreased muscle mass and motor function, but no significant differences between CC and ASM were caused by obesity. This was supported by the results of our fitness factor analysis based on SAF level. Physical activity, which is essential for preventing sarcopenia, was also associated with SAF. Repetitive physical activity plays a significant role in reducing the formation of reactive oxygen species and preventing the accumulation of AGEs [25]. Previous studies have reported decreases in SAF with increasing levels of regular physical activity [26]. In this study, as SAF increased, we found significant differences in other key indicators of sarcopenia such as CC, DHG, SPPB, and gait speed. Our multiple regression analysis fitness factor model proved to be able to predict sarcopenia from SAF measurements.

Nevertheless, this study has several key limitations worth noting. First, it was conducted only in participants aged ≥60 years living in Incheon, South Korea—where normal SAF ranges have not yet been defined. Second, the fitness factor was analyzed but sarcopenia was not analyzed individually; therefore, it could not be identified, and other variables related to obesity were not considered. Further studies are therefore warranted to confirm the relationship between physical activity and sarcopenia, chronic diseases, and SAF. We also consider the establishment of SAF reference values for the Korean population to be crucial.

This study could confirm the role of SAF as a potential biomarker for chronic diseases such as diabetes, hyperlipidemia, and hypertension. Our results suggested that SAF may also be useful for predicting chronic diseases associated with sarcopenia. However, our study still had some limitations. Since this study did not investigate the specific requirements of the target population or provide customized interventions, further studies are warranted to validate these results in other populations, as well as to establish clear normal or baseline SAF values in South Korea. Our findings have potential implications for advancing preventive and diagnostic approaches in geriatric medicine, although further studies should be conducted to verify the full extent of the associations between the roles of SAF and the variables it affects.

## 4. Materials and Methods

### 4.1. Participants

This study included older adults aged 60–100 years living in Incheon, Republic of Korea. The participants comprised 565 (129 men and 436 women) users of facilities for older adults, including welfare and protection centers. Before the study, all participants were provided a detailed description of the purpose, methods, and risks involved; they were also informed that they could withdraw from the experiment at any time without repercussions. All participants included in the study cohort signed an informed consent form. All study procedures were approved by the Ga Chon University Institutional Bioethics Committee (approval no. 1044396-202309-HR-188-01). This study was conducted following the principles of the Declaration of Helsinki.

### 4.2. Study Design

The study was conducted from March to June of 2023 among the users of 12 welfare and protection centers in Incheon, South Korea. Before the measurements, a survey was conducted on the levels of physical activity, chronic disease, or health problems of all participants. The noninvasive evaluation of SAF was performed utilizing an AGE Reader mu (Diagnoptics Technologies, Groningen, The Netherlands), which employs excitation light with a peak wavelength of around 375 nm directed onto the skin. SAF values were computed by the device as the ratio of the emitted light (420–560 nm) from the fluorescent molecules in the skin to the reflected excitation light (300–420 nm), multiplied by 100, expressed in arbitrary units (AU), and adjusted for low skin reflectance—with reflectance indicating the corrected reflectance at 375 nm [27]. HbA1c (mg/dL) was measured using a blood glucose meter (GC Med Sci, Seoul, Republic of Korea).

**Figure 2:**
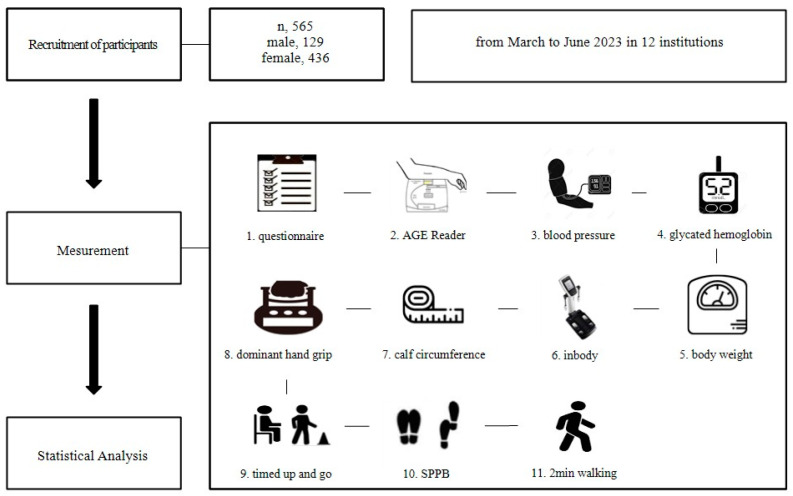
Study design.

The fitness factor was evaluated in the same location immediately following the SAF assessment. The evaluation included a series of standardized measurements: body composition was assessed using a multi-frequency bioelectrical impedance analyzer (InBody), while blood pressure was recorded in a seated position using an automated oscillometric device after a minimum of five minutes of rest. Calf circumference (cm) was measured at the largest girth of the calf with a non-elastic tape, and body weight (kg) was measured using a calibrated digital scale. The handgrip strength (kg) of the dominant hand was measured using a hydraulic dynamometer, adhering to established testing protocols. Functional mobility was assessed via the timed up-and-go (TUG) test by timing the participant as they stood from a chair, walked three meters, turned, returned, and sat down. Lower extremity function was further evaluated through the short physical performance battery (SPPB), including balance, gait speed, and repeated chair stands. Lastly, aerobic endurance was assessed with the 2-minute step test, during which the number of knee raises to a specified height while stepping in place was recorded over two minutes (Figure 2).

### 4.3. Statistical Analysis

All results were presented as the means ± standard deviations. Statistical analysis was conducted using SPSS version 26.0 (SPSS Inc., Chicago, IL, USA). The differences between groups were assessed using one-way analysis of variance (ANOVA), followed by Tukey’s test for post hoc analysis. Principal component analysis was employed alongside multiple logistic regression for model analysis between variables. Statistical significance was determined at *p* < 0.05.

## Figures and Tables

**Table 1:** The demographic characteristics of the participants.

Variables		Male	%	Female	%
Age (years)	60–69	24	18.6	101	23.2
	70–79	43	33.3	167	38.3
	80–89	55	42.6	149	34.2
	<90	7	5.5	19	4.3
Height (cm)	~155.9	26	20.2	412	94.5
	160–164.9	45	34.9	20	4.7
	165–169.9	30	23.2	3	0.6
	<170	28	21.7	1	0.2
Weight (kg)	~44.9	3	2.3	40	9.2
	45–54.9	13	10.1	153	35.1
	55–64.9	49	38.0	165	37.8
	65–74.9	42	32.5	52	11.9
	<75	22	17.1	26	6.0
Body fat mass (%)	~19.9	4	3.1	24	5.5
	20–29.9	32	24.8	99	22.7
	30–39.9	51	39.6	217	49.8
	<40	42	32.5	96	22.0
BMI (kg/m^2^)	~18.5	3	2.3	17	3.9
	18.6–24.9	81	62.9	231	53.0
	25–29.9	42	32.5	152	34.8
	<30	3	2.3	36	8.3
ASM (kg)	~5.9	5	3.9	239	54.8
	6–8.9	120	93.0	195	44.7
	<9	4	3.1	2	0.5
Diabetes	Yes	43	33.3	131	30.1
	No	86	66.7	305	69.9
Hypertension	Yes	62	48.1	240	55.0
	No	67	51.9	196	45.0
Hyperlipidemia	Yes	38	29.5	173	39.7
	No	91	70.5	263	60.3
total		129	100	436	100

BMI, body mass index; ASM, appendicular skeletal muscle.

**Table 2:** Odds ratio (95% confidence interval) for SAF stage and factor variables.

Variable Category		Male = 129		Female = 436	
		OR (95% CI) and *p*			
HbA1c (mg/dL)	≤2.4	Reference			
	2.5–2.8	0.920 (0.457~1.854)	0.056	0.968 (0.710~1.320)	0.839
	2.9–3.3	1.094 (0.564~2.124)	0.079	1.111 (0.824~1.498)	0.491
	>3.4	0.807 (0.393~1.657)	0.598	1.878 (1.411~2.501) ***	<0.001
ASM (kg)	≤2.4	Reference			
	2.5–2.8	1.671 (0.456~6.128)	0.439	1.129 (0.735~1.735)	0.580
	2.9–3.3	2.759 (0.731~10.419)	0.134	1.021 (0.661~1.579)	0.924
	>3.4	1.982 (0.551~7.121)	0.295	0.637 (0.378~1.076)	0.092
CC (cm)	≤2.4	Reference			
	2.5–2.8	0.706 (0.492~1.013)	0.059	0.978 (0.848~1.127)	0.755
	2.9–3.3	0.680 (0.468~0.987) *	0.043	0.913 (0.791~1.055)	0.217
	>3.4	0.647 (0.451~0.926) *	0.017	1.088 (0.918~1.288)	0.331
DHG (rep)	≤2.4	Reference			
	2.5–2.8	0.893 (0.788~1.013)	0.079	0.933 (0.876~0.994) *	0.032
	2.9–3.3	0.949 (0.828~1.087)	0.451	0.956 (0.898~1.019)	0.164
	>3.4	0.842 (0.740~0.958) **	0.009	0.965 (0.899~1.036)	0.325
SPPB (rep and sec)	≤2.4	Reference			
	2.5–2.8	2.179 (1.177~4.033) *	0.013	1.549 (1.291~1.858) ***	<0.001
	2.9–3.3	1.758 (0.951~3.250)	0.072	1.496 (1.250~1.789) ***	<0.001
	>3.4	2.110 (1.159~3.839) *	0.015	1.541 (1.266~1.875) ***	<0.001
TUG (rep)	≤2.4	Reference			
	2.5–2.8	1.355 (0.878~2.090) *	0.017	1.161 (0.971~1.193)	0.161
	2.9–3.3	1.683 (1.064~2.660) *	0.026	1.053 (0.950~1.167)	0.327
	>3.4	1.433 (0.936~2.194)	0.098	1.073 (0.966~1.191)	0.188
2 min walking test (rep)	≤2.4	Reference			
	2.5–2.8	0.984 (0.951~1.018)	0.343	0.989 (0.977~1.001)	0.065
	2.9–3.3	0.968 (0.936~1.002)	0.065	0.987 (0.976~0.999) *	0.035
	>3.4	0.962 (0.931~0.995) *	0.022	0.979 (0.966~0.992) **	0.002
Gait speed (sec)	≤2.4	Reference			
	2.5–2.8	1.025 (0.523~2.009)	0.942	1.071 (0.837~1.370)	0.585
	2.9–3.3	0.503 (0.228~1.107)	0.088	1.103 (0.863~1.410)	0.433
	>3.4	0.837 (0.434~1.614)	0.595	1.118 (0.869~1.439)	0.387

Significant difference: * *p* < 0.05, ** *p* < 0.01, *** *p* < 0.001; tested by performing multiple logistic regression analysis. SAF, skin autofluorescence; OR, odds ratio; CI, confidence interval; ASM, appendicular skeletal muscle; CC, calf circumference; DHG, dominant hand grip; SPPB, SPPB score; TUG, timed up-and-go.

**Table 3:** Odds ratios with 95% confidence intervals for the factors on SAF risk.

Variable Category		(a) Model 1		(b) Model 2		(c) Model 3	
		OR (95% CI)	*p*	OR (95% CI)	*p*	OR (95% CI)	*p*
SAF	≤2.4	Reference					
	2.5–2.8	1.008 (0.999~1.017)	0.073	1.346 (0.522~3.471)	0.239	1.002 (0.998~1.005)	0.539
	2.9–3.3	1.006 (0.996~1.015)	0.239	2.154 (0.879~5.294)	0.239	1.001 (0.997~1.005)	0.669
	>3.4	1.018 (1.009~1.027)	<0.001 ***	2.639 (1.054~6.608)	0.038 *	1.004 (1.000~1.007)	0.035 *

Significant difference: * *p* < 0.05 and *** *p* < 0.001; tested by performing multiple logistic regression analysis. (a) Model 1, further adjusted for age and sex; (b) Model 2, further adjusted for disease factor (diabetes, hypertension, hyperlipidemia); (c) Model 3, further adjusted for fitness factor (timed up-and-go, gait speed); SAF, skin autofluorescence; OR, odds ratio; CI, confidence interval.

## Data Availability

The datasets used and/or analyzed during the current study are available from the corresponding author on reasonable request.

## Abbreviations

The following abbreviations are used in this manuscript:

AGE	Advanced glycation end product
ANOVA	Analysis of variance
AMPK	Activated protein kinase
ASM	Appendicular skeletal muscle
AU	Arbitrary units
BMI	Body mass index
CC	Calf circumference
DHG	Dominant hand grip
DM	Diabetes mellitus
HbA1c	Glycated hemoglobin
GLUT-4	Glucose transporter-4
IL-6	Interleukin 6
NFκB	Nuclear factor kappa B
MuRF1	Muscle RING-finger protein-1
PI-3	Phosphoinositide-3
SAF	Skin autofluorescence
SPPB	Short physical performance battery
TNF-α	Tumor necrosis factor-α
TUG	Timed up-and-go
